# Fixing science means an end to gaming the system

**DOI:** 10.1371/journal.pbio.3002816

**Published:** 2024-09-09

**Authors:** Nicholas J. L. Brown

**Affiliations:** Department of Psychology, Linnaeus University, Växjö, Sweden

## Abstract

During the last decade, there has been a substantial acceleration in the open science movement. Most people would probably hope to have seen signs of positive change in that time, yet it seems that the process of improving the practice of science is moving at a glacial pace.

## Abstract 2

The last decade has seen a substantial acceleration in the open science movement, yet it seems that the process of improving the practice of science is moving at a glacial pace. This Perspective explores the misaligned incentives that are hindering progress to more open, reproducible research.

Although the issue of the (lack of) reproducibility of scientific research had been raised before, for example in the context of cancer, the May 2014 special issue of *Social Psychology* was a first for scientific publishing. It contained exclusively Registered Reports, a publishing format in which, unlike traditional research articles, the proposed methodology for a research study is peer-reviewed prior to data collection and the study is published if the methodology is adhered to, independently of the nature of the results. Its editorial [1] highlighted the poor reproducibility of a lot of published social science. This was also probably the first time when calls to make scientific publication more transparent received direct pushback [2] from authors who perceived themselves to have been critiqued. Since 2014, over 350 journals have adopted Registered Reports, and there has been a surge of interest in the wider open science movement, notably among younger researchers. And yet, reading most journals 10 years later, it does not feel to me as if the reality of scientific publishing has changed very much. Registered Reports remain the exception as a publication format, and many of the claims by authors to have adopted open science practices often turn out to be merely window-dressing.

We should acknowledge substantial progress in at least 2 areas. First, the tools for making one’s research transparent, such as the Open Science Framework and reproducibility-promoting software like Rmarkdown, have become readily available and are easy to use. Second, there has been a great deal of effort in raising awareness of the desirability of performing one’s research in a transparent way. There is no doubt that being seen to “do open science” is the cool place to be right now [3].

But while one can lead scientists to the pure water of best practices, it can be hard to get them to drink, especially when tastier metaphorical beverages are still available on tap. Open science arose in response to the realisation that scientists were churning out too much junk, but junk seems to be what the “market” that interests scientists—the one in which they find publications, prestige, and tenure—still demands. Merely publishing the data set of your p-hacked [4] study is a bit like replacing the white bread in your diet with an equivalent amount of whole wheat, while continuing to consume 3 litres of sugary soft drinks per day.

We are already starting to see some authors and publishers gaming laudable open science initiatives in order to gain the credibility benefits of “being open” while not actually changing very much. One egregious example of this gaming is the case of authors earning a “Preregistered” badge for their entire paper even if the preregistration contains minimal detail, or applies to only one of several reported studies; indeed, the journal *Psychological Science*, which was one of the first to introduce this badge, has now discontinued its use, at least in part because of this issue [5]. In another case, a field-leading journal rejected a completed Stage 2 Registered Report on the basis of the results obtained after pre-registration, defeating the purpose of this article type. A third example is the data sharing policy of the entire JAMA network of journals [6]—which, while paying lip service to openness by requiring authors of articles to provide a “data sharing statement” allows that statement to consist of “we will not share our data” with no requirement to give reasons. Even the journal *PLOS ONE*, a pioneer of supposedly mandatory data sharing, has taken no action beyond a seven-year-old expression of concern [7] for a failure to share data from an article [8] that continues to have substantial influence on clinical practice worldwide. The mentality still seems to be “How can I get published?” far more often than “What is true?”.

The fundamental problem, which also led to the situations that the open science movement has tried to address, is that what is good for science is not necessarily good for the individual scientists who are involved in its production. Indeed, in some ways those interests are often diametrically opposed, especially in view of the economic realities of the societies in which most scientists live. This situation has not changed markedly in the past 10 years. Recruiting larger samples to reduce the possibility of statistical flukes, and designing our studies in ways that actually constrain our ability to change our minds about what we were looking for, are not conducive to the “productivity” that is still widely expected of scientists by their institutions, despite initiatives such as the San Francisco Declaration on Research Assessment (SF-DORA). Until we genuinely re-align these incentives, gaming will continue to be the economically rational thing to do.

Giving up gaming will not be easy. People do not like being asked to work harder for less reward, and especially (though not only) for senior people, being confronted with the fact that their own research careers may have been built on sand may require them to stare into the abyss. But without this frank introspection, we risk looking at ourselves in the mirror in another 10 years and discovering once again that nothing much has changed. In the last few years, a number of highly respected open science proponents have left academia, and I know that in several cases this was due to a belief that cutting corners is still the best way to get ahead. We will not be making progress until the people who are leaving are those who realise that they can no longer get away with not putting in the hard effort required to do science more robustly and transparently.

I do not have an easy solution to offer to this problem. My feeling is that it will be hard to address without some kind of external supervision, but I do not pretend to know how this could be done at scale while avoiding political interference, and also given the transnational nature of science. Perhaps the aviation industry has something to teach us here. People dislike airport security, but everyone knows that the rules apply to them; if the machine goes beep, you’re going to be patted down even if you have a first-class ticket, and if you refuse to show the contents of your luggage, you’re not going to be allowed to board the plane. Meanwhile, on the operational side, aviation uses a system of good-faith investigations of near-misses in which establishing the truth is prioritised and all parties are encouraged to speak up without fear of reprisals. If nothing is done, I fear that at some stage we might need the academic equivalent of the type of Truth and Reconciliation Commission that has been used in some countries to “wipe the slate clean” in the aftermath of armed civil conflicts.

Despite these difficulties, I think it is important to have this type of discussion, if only so that if we decide that we are not prepared to do anything about gaming, we do so *consciously*. For what it’s worth, I think that the chances of serious, hard-to-game measures such as Registered Reports—which are probably the single most effective way to reduce publication bias [9]—becoming the normal way of doing all science within the next 10 years are well below 50%. Of course, it’s possible that the supertanker of science is in fact very slowly turning and that we haven’t noticed it yet, but which of us has the time to wait to find that out?

## References

[1] Nosek BA, Lakens D. Registered Reports: A method to increase the credibility of published results. Soc Psychol. 2014;45:137. doi: 10.1027/1864-9335/a000192

[2] Schnall S. An experience with a registered replication project. 2014 May 22 [cited 2024 July 31]. Available from: https://www.psychol.cam.ac.uk/cece/blog.

[3] Nature. Open science—embrace it before it’s too late. Nature. 2024;626:233. doi: 10.1038/d41586-024-00322-2 38321141

[4] Simmons JP, Nelson LD, Simonsohn U. False-positive psychology: Undisclosed flexibility in data collection and analysis allows presenting anything as significant. Psychol Sci. 2011;22:1359. doi: 10.1177/0956797611417632 22006061

[5] Hardwicke TE, Vazire S. Transparency is now the default at Psychological Science. Psychol Sci. 2024;35:708. doi: 10.1177/09567976231221573 38150599

[6] Flanagin A, Curfman G, Bibbins-Domingo K. Data sharing and the growth of medical knowledge. JAMA. 2022;328:2398. doi: 10.1001/jama.2022.22837 36469324

[7] The *PLOS ONE* Editors. Expression of Concern: Adaptive pacing, cognitive behaviour therapy, graded exercise, and specialist medical care for Chronic Fatigue Syndrome: A cost-effectiveness analysis. PLoS ONE. 2017;12:e0177037. doi: 10.1371/journal.pone.0177037 28463341 PMC5412692

[8] McCrone P, Sharpe M, Chalder T, Knapp M, Johnson AL, Goldsmith KA, et al. Adaptive pacing, cognitive behaviour therapy, graded exercise, and specialist medical care for Chronic Fatigue Syndrome: A cost-effectiveness analysis. PLoS ONE. 2012;7:e40808. doi: 10.1371/journal.pone.0040808 22870204 PMC3411573

[9] Scheel AM, Schijen MRMJ, Lakens D. An excess of positive results: Comparing the standard psychology literature with Registered Reports. Adv Methods Pract Psychol Sci. 2021;4:1. doi: 10.1177/25152459211007467

